# Graph theoretical comparison of functional connectivity between *cLTP *treated and untreated microelectrode arrays

**DOI:** 10.1186/1471-2202-16-S1-P90

**Published:** 2015-12-18

**Authors:** Myles Akin, Rhonda Dzakpasu, Yixin Guo

**Affiliations:** 1 Department of Mathematics, Drexel University, Phildelphia, PA, 19104, USA; 2 Department of Physics, Georgetown University, Washington, DC 20057, USA

## 

Analyzing graph properties of neural networks has recently gained much attention in attempts to understand how information is processed in the brain. Using *in-vitro *techniques to form neural networks has increased in popularity as it allows one to develop small, easy to record networks that maintain many of the graph properties of larger brain networks [1]. One widely recognized tool for studying *in vitro *networks is the Microelectrode Array (MEAs) on which neurons can be cultured and recorded simultaneously. MEAs can be used to grow neural networks from disassociated cells to understand how neurons spontaneously connect to create networks and how these networks then evolve over time. In addition, these cultures can be treated with pharmacological agents to study how these agents affect the networks as a whole [2,3].

To understand the network formation of MEA cultured neurons, we study the graph theoretical properties of two MEAs networks, the control MEA network and the MEA network treated with chemical Long Term Potentiation (cLTP). The data sets for each MEA network consists of recording from three days: baseline, 2 days past baseline and 5 days past baseline. Based on these data sets and the assumption that each electrode on the MEA records one neuron, we construct functional connectivity graphs of MEA networks for different days. Nodes in such a connectivity graph represent the electrodes (also neurons). To determine whether there is a connection (an edge on the graph) between two nodes, we carry out several steps of computations. We first filter the recorded spike trains with a Gaussian kernel, and perform cross-correlation analysis using the Pearson product moment correlation coefficient [4]. We set a correlation threshold by applying a shuffling method to the inter-spike intervals of a spike train.

**Figure 1:**
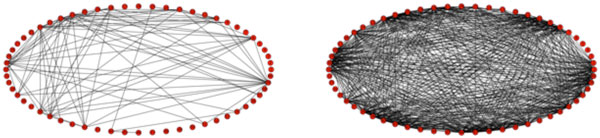
**Graph models**. (a) cLTP treated MEA network at baseline; (b) cLTP treated MEA network at 5 days past baseline.

Using thresholded correlations, unweighted, undirected adjacency matrices, we create corresponding graphs for untreated (not shown) and treated MEA networks (shown baseline and 5 days past baseline in Figure 1). We find that the synchronization and average node degree increase dramatically for the cLTP treated networks while the untreated network shows no obvious change.

To better understand the treated and untreated MEA network, we will evaluate the graph theoretic properties, such as degree distribution and clustering coefficient. We will determine how cLTP affects these properties. The graphical analysis will enable us to identify what type of network each is (such as a small-world or a scale free network) and determine whether cLTP has an effect on the network development or merely on the strength of connectivity. We conjecture that cLTP treated networks have more efficient and quicker communication between nodes. Therefore, the cLTP treated networks show greater clustering as well as shorter path length than the untreated networks. Information flow is another important aspect of such graph model. We intend to develop directed graphs using transfer entropy to study how information flow of the network may change during its development.
